# Correspondence

**Published:** 1912-04

**Authors:** J. M. Higgins

**Affiliations:** Arizpe, Sonora, Mexico


### CORRESPONDENCE

Arizpe, Sonora, Mexico,
February 23, 1912.

Editor BUFFALO MEDICAL JOURNAL:

I received copy of JOURNAL requested and will send in my subscription just as soon as our post office is allowed to issue money orders again. We are having lots of trouble lately with Revolutionists and it is difficult to get money through the mails. However, I will send in my subscription as soon as possible. Your JOURNAL is one of the best for the practitioner, printed. Thanking you for your prompt reply, I remain

Yours respectfully,

J. M. HIGGINS.

(Note: While the BUFFALO MEDICAL JOURNAL aims especially to serve as a local medium for western New York, we frequently receive orders from quite distant points, including Central and South American countries, South Africa, etc. Some of these orders, it must be confessed, come from professional "samplers" in our own country but most of them are bona fide and fully half are from men who, so far as we can discover, have no local affiliations. We hope later to hear some of Dr. Higgin's experiences.)

